# High-Value Utilization of Amaranth Residue and Waste LDPE by Co-Pyrolysis

**DOI:** 10.3390/molecules30173471

**Published:** 2025-08-23

**Authors:** Julia Karaeva, Svetlana Timofeeva, Svetlana Islamova, Marina Slobozhaninova, Ekaterina Oleynikova, Olga Sidorkina

**Affiliations:** Institute of Power Engineering and Advanced Technologies, FRC Kazan Scientific Center, Russian Academy of Sciences, 420111 Kazan, Russia; zvezdochka198512@mail.ru (S.T.); leylik.sidorkina@mail.ru (O.S.)

**Keywords:** utilization, co-pyrolysis, waste, amaranth residue, pyrolysis liquid, biochar

## Abstract

Amaranth is important for the agro-industrial complex. However, when extracting flour and oil from seeds, a lot of waste remains. Waste recycling by co-pyrolysis aims at obtaining new products with high added value. This study examined a combination of *A. cruentus* (AC) residues and low-density polyethylene (LDPE) waste. The addition of polymer was aimed at obtaining hydrocarbon-rich pyrolysis liquid and biochar. Pyrolysis was performed on an experimental setup, along with thermogravimetry–Fourier infrared spectroscopy–gas chromatography mass spectrometry (TG-FTIR-GC MS), to examine the thermochemical conversion. Experiments were carried out using a thermogravimetric analyzer at heating rates of 5, 10, and 20 °C/min. The average activation energy values for the pyrolysis of the AC/LDPE blend by the Ozawa–Flynn–Wall (OFW) and Kissinger–Akahira–Sunose (KAS) techniques were 301.39 kJ/mol and 287.69 kJ/mol, respectively. A visual examination of the correlations of the kinetic parameters of AC/LDPE was carried out using the Kriging method. The pyrolysis liquid from AC contains 38.14% hydrocarbons, with the main part being aliphatic hydrocarbons. During the pyrolysis of the AC/LDPE mixture, hydrocarbons were found in the resinous and waxy organic fractions of the pyrolysis liquid. The composition and properties of AC and AC/LDPE biochar are similar, and they can both be applied to agriculture.

## 1. Introduction

Amaranth is a unique plant of great importance for food security. In addition, it is a promising pseudocereal crop rich in protein (13–22%) and lysine (3.2–6%) [1]. Amaranth grain is a rich source of squalene, so it is used to produce oil for both edible and pharmaceutical purposes. The use of this plant in modern agriculture is explained by its resistance to various climatic conditions [2]. Amaranth can grow in a wide range of weather conditions, and it is characterized by drought resistance. Because the plant cannot be stored for a long time, a large amount of amaranth ends up as waste, which leads to economic losses and creates environmental problems [3]. Of the approximately 70 species, only *A. hypochondriacus*, *A. cruentus*, and *A. caudatus* are used to produce edible grain [4]. Large amounts of biomass remain after grain harvesting, representing an agro-industrial waste with high potential for producing new high-value-added products. It should be noted that there are few studies in the literature that have been devoted to the valorization of amaranth biomass.

Thermochemical conversion enables waste to be processed into high-value-added products. One of the most modern methods is co-pyrolysis. This method is becoming increasingly known as an economical way to convert a variety of raw materials into energy and high-quality goods [5].

Mixing biomass and plastic waste as feedstock for the co-pyrolysis process has become economically feasible for a number of reasons [6,7]: the resulting pyrolysis liquid will have lower oxygen content, moisture content, corrosive activity, and viscosity; the addition of plastic can help to increase the yield and quality of the pyrolysis liquid and gas; the resource cycles of biomass and plastic waste are closed in one technological chain for the production of biofuels; and it allows for scalability.

When compared to individual pyrolysis, the majority of research findings show that co-pyrolysis has synergistic effects [8]. In the co-pyrolysis of a mixture of *Pongamia pinnata* seeds and plastic waste, for instance, the average activation energy values decreased, suggesting a lower energy need [9].

Because of its unique molecular chain structure and high hydrogen-to-carbon ratio of about two, low-density polyethylene (LDPE) is the best plastic for creating liquid hydrocarbons or fuels [10]. According to a literature review, polymer components make up for biomass’s natural lack of hydrogen. Consequently, the addition of a hydrogen donor or free radical modifies the properties of the ultimate biomass product and controls the composition of intermediate products. Furthermore, the carbon produced during biomass pyrolysis can degrade plastic by acting as a catalyst for processes. At various pyrolysis temperature stages, the pyrolysis of polymers and biomass produces intermediate products and free radicals. The distribution and properties of the products are influenced by the interactions between the various free radicals.

The potential to produce pyrolysis liquid rich in aliphatic compounds was demonstrated by the successful co-pyrolysis of cedar wood, sunflower stalks, and *Fallopia japonica* stems with LDPE [11]. It was previously established that the pyrolysis liquid from the inflorescences of *A. cruentus* contains a large amount of hydrocarbons [12]. Thus, the addition of LDPE to amaranth waste can potentially enrich the resulting liquid product. The very high volatile content of LDPE will also have a minimal impact on the composition of the resulting biochar.

The majority of research that evaluated the effectiveness of co-pyrolysis [13] used the thermogravimetric analysis (TGA) method [14,15,16,17] and kinetic analysis based on TGA data [18,19,20,21,22]. It should be noted that a small number of studies were devoted to the study of co-pyrolysis in a pyrolyzer [23,24]. There are data on the combined pyrolysis of various types of biomass and LDPE [14,15,16,17], but there are no data on the use of amaranth. The study of chemical kinetics using thermal analysis methods and obtaining the necessary kinetic parameters are important for optimizing the pyrolysis process and designing industrial equipment.

Thermal analysis, in addition to determining the amount of mass loss, allows one to obtain the kinetic parameters of the pyrolysis process. To assess the kinetics of the thermal decomposition of biomass, plastics, and their mixtures, model-free methods (Ozawa–Flynn–Wall (OFW), Kissinger–Akahira–Sunose (KAS), and Friedman) are most often used [18,19,20,22,25]. Currently, the visual examination of the correlations of the kinetic parameters of thermochemical conversion is of great interest. For these purposes, the statistical response surface methodology is often used. However, to solve high-order nonlinear problems, it is better to use the Kriging regression method [26]. In addition, for the pyrolysis process, it is proposed for the first time to use normalized values to analyze the correlations of the parameters.

The purpose of this study is to experimentally investigate the co-pyrolysis of amaranth residues (the above-ground parts of plants—leaves, stems, and inflorescences) and LDPE waste, as well as conducting a detailed study of the prospects for processing to obtain new liquid and solid products with high added value. Thermogravimetry–Fourier infrared spectroscopy–gas chromatography mass spectrometry (TG-FTIR-GC MS) was used to comprehensively evaluate the pyrolysis characteristics and pyrolysis liquid. While previous studies have focused on process kinetics and product characteristics, this study will also attempt to reveal any correlations among three kinetic factors (pre-exponential factor *A*, activation energy *E_α_*, and algebraic function *g*(*α*)). The kinetic analysis was complemented by a visual examination of the correlations of the kinetic triplet parameters using the Kriging method. This integrated approach contributes to a deeper understanding of thermochemical conversion processes and is important for filling some gaps in the scientific literature.

## 2. Results

### 2.1. Results of Proximate and Ultimate Analyses

The physicochemical properties of the *A. cruentus* (AC), LDPE, and AC/LDPE samples are shown in Table 1. The elemental composition of AC and LDPE is comparable with the values characteristic of lignocellulosic materials [27] and polymers [28], respectively. All indicators are given for the air-dried state of the samples.

The moisture content of the samples is less than 10 wt.%, on an air dry basis, which contributes to an increase in the thermal efficiency of the process and to an improvement in the quality of the products. In addition, the samples have a high content of volatile matters (75.8 wt.%, on an air dry basis, and higher) and a sufficiently high HHV, which makes them suitable for the pyrolysis process [29,30]. The high content of volatile matters characterizes the high reactivity of the material, which contributes to pyrolysis with a high yield of gaseous and liquid components. The ash content of AC is 13.0 wt.%, on an air dry basis, and this is quite high for biomass.

### 2.2. TGA Results

**Figure 1:**
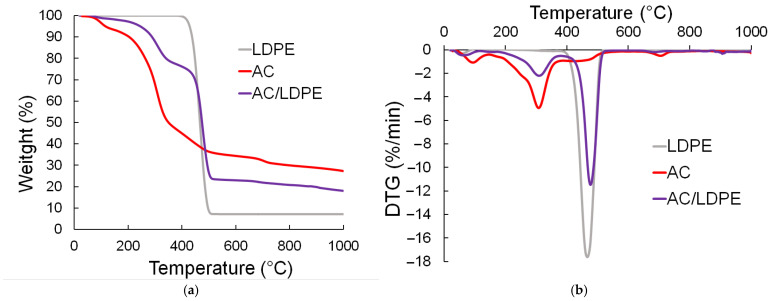
(**a**) TG curves; (**b**) DTG curves.

**Figure A1:**
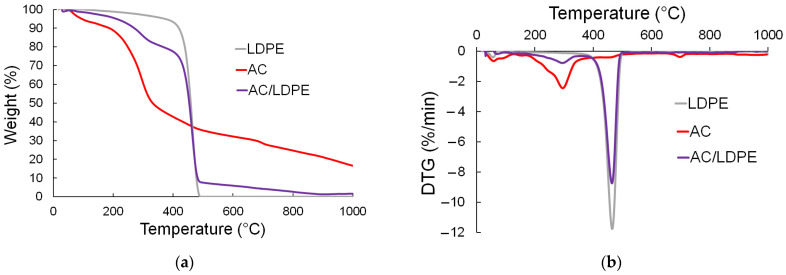
Heating rate of 5 °C/min: (**a**) TG curves; (**b**) DTG curves.

**Figure A2:**
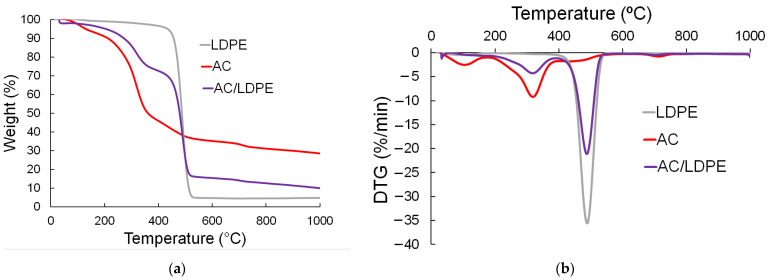
Heating rate of 20 °C/min: (**a**) TG curves; (**b**) DTG curves.

Depending on the temperature, the TG and derivative thermogravimetric (DTG) curves are created. DTG curves display the weight loss brought on by phase transitions during pyrolysis reactions. In contrast to biomass, which decomposes in three stages—drying, active, and passive pyrolysis—plastic waste typically decomposes in a single stage [31,32,33,34]. Figure 1 shows the TG and DTG curves of the samples and their mixture at a heating rate of 10 °C/min. The TG and DTG curves at heating rates of 5 and 20 °C/min are presented in Appendix A as Figure A1 and Figure A2.

The structural variations in the constituents and their combinations that comprise the materials under study are the reason why the AC/LDPE co-pyrolysis curves demonstrate distinct thermal behavior patterns. The three main stages of weight loss during the thermal decomposition of the AC/LDPE blend are as follows: fast decomposition in the relevant temperature range (from about 190 °C to 550 °C), slow decomposition caused by the secondary decomposition of the components at high temperatures above 550 °C, and the loss of bound moisture (from ambient temperature to about 190 °C). The primary temperature ranges that define the stages of thermal decomposition are shown in Table 2.

The drying stage, which is linked to the biomass’s residual moisture content, is normal for the AC and AC/LDPE samples [35,36,37,38,39]. The initial stage of moisture removal occurs when the LDPE sample is heated, due to its high hydrophobicity. Furthermore, at this point, mild volatile components might be emitted.

**Figure 2:**
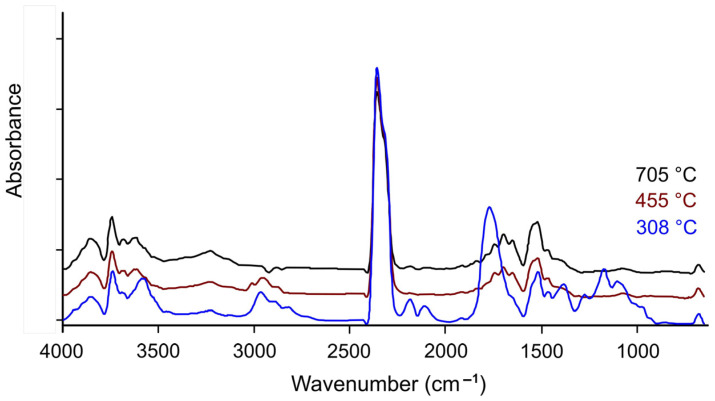
FTIR spectrum of gaseous products for AC.

The fast decomposition of biomass components and the emission of volatile compounds linked to the thermal breakdown of cellulose, hemicellulose, and lignin took place during the active pyrolysis stage. The fast decomposition of the AC samples resulted in an average mass loss of 64.9%. One may observe temperature peaks with values of 319 and 434 °C on the DTG curves (Figure 2). Furthermore, a slight temperature exothermic peak is found around 400 °C, which can be attributed to the start of the sample’s lignin breakdown. Over 550 °C, the rate of weight loss progressively decreased, indicating that the second stage of decomposition had nearly finished releasing the volatile chemicals.

Pure LDPE was destroyed in a single step throughout the experiment’s polymer’s heat deterioration. The plastic started to decompose at around 335 °C and went through one phase at a higher temperature within a specific range. The lengthy chain structure of polymeric polymers was the cause of this. Up to 520 °C, LDPE lost roughly 92.7% of its mass; beyond that, it did not break down. The mass of the resultant residue was 7.12% (Table 3).

The TG and DTG curves also demonstrate how the constituent components affected the structural characteristics of the AC/LDPE blends. The character of the curves and the overall pattern of thermal degradation match the results from the co-pyrolysis of blends of biomass and plastic waste [40,41,42]. The sample’s residual mass was 18.1%.

The slow decomposition stage is observed in the AC and AC/LDPE samples. At this stage, after the release of volatile components and completion of the main thermal destruction, the thermal decomposition of the inorganic components of the biomass occurs. First, CaCO_3_ enters the process of the thermal destruction of mineral components, which decompose in the temperature range from 780 °C to 1000 °C [31].

### 2.3. FTIR Spectrum Analysis

**Figure 3:**
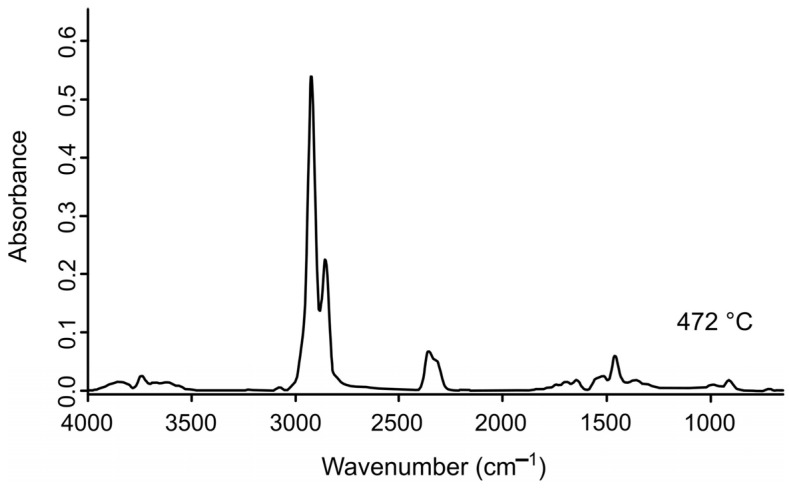
FTIR spectrum of gaseous products for LDPE.

**Figure 4:**
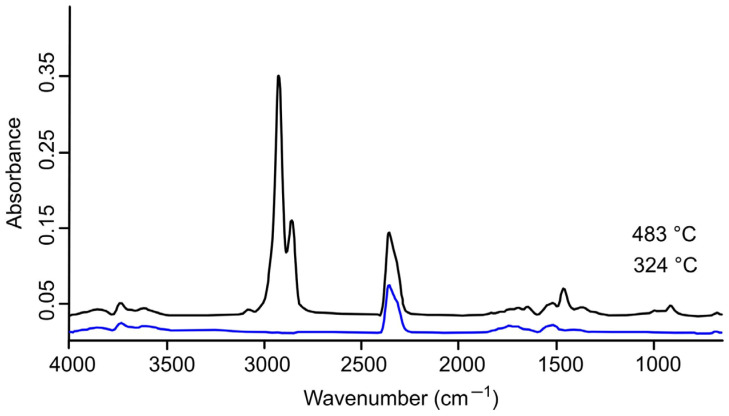
FTIR spectrum of gaseous products for AC/LDPE.

The volatiles released during the pyrolysis of the AC, LDPE, and AC/LDPE samples were analyzed in real time using FTIR to determine the structure of the functional groups of the resulting gaseous substances from the characteristic infrared peaks [20]. Figure 2, Figure 3 and Figure 4 show the IR signals of CO_2_ (2355, 2356 cm^−1^); CO (2108 cm^−1^, 2181 cm^−1^); H_2_O (4000–3400 cm^−1^, 2000–1250 cm^−1^); and aromatic compounds (–C–H bond bending vibration at 1515–1519 cm^−1^), aliphatic compounds (–C–H bond bending vibration at 1458 cm^−1^ and –C–H bond stretching vibration at 2858, 2860, 2926, 2928 cm^−1^ and 2926, 2928 cm^−1^; –C=C bond bending vibration at 909, 910 cm^−1^ and stretching vibration at 1645 cm^−1^ and 1695, 1696 cm^−1^, and 1771 cm^−1^), and alcohols/ketones/esters (–C=O bond stretching vibration at 1742 cm^−1^ and 1771 cm^−1^ and 3586, 3610, 3628 cm^−1^) [20,43,44,45,46].

Significant volumes of water vapor are produced during the pyrolysis of AC due to the breakdown of oxygen-containing groups (Figure 2). At low temperatures, the pyrolysis of hemicellulose is thought to be the primary source of CO_2_ [46]. The breakdown of carboxyl (C–O–C) and carbonyl (C=O) with low thermal stability is the primary cause of the little CO release that is seen at 308 °C [20]. Numerous functional group vibrations, such as –O–H stretching, –C–H stretching in aliphatic and aromatic compounds, and –C=O, –C=C, and –C=O stretching, can be observed at each temperature (308, 455, and 705 °C).

The IR spectrum recorded for the vapor products of LDPE pyrolysis at 472 °C is shown in Figure 3. The dominance of the CH stretching vibration signal in the spectrum indicates that the gaseous pyrolysis products are rich in hydrocarbons [47]. For CO_2_, noticeable small peaks are observed for water vapor and aromatic products (–C=C–).

As the AC/LDPE combination was heated in the pyrolysis process, spectral oscillations were captured between 324 °C and 483 °C (Figure 4).

The main absorption peak here is also the stretching of the C–H bond, indicating the production of a significant amount of aliphatic hydrocarbons. There are small oscillations in the spectra of aliphatic and aromatic compounds as weak signals are present. The CO_2_ peak is dominant at 324 °C. At 483 °C, it is still strong, only next to the C-H peaks, which is anticipated since LDPE pyrolysis products are rich in aliphatic hydrocarbons.

### 2.4. Kinetic Analysis

#### 2.4.1. Model-Free Methods

**Figure 5:**
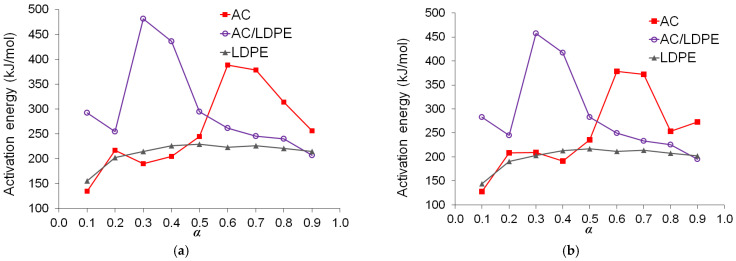
Values of *E_a._* for each degree of conversion: (**a**) OFW; (**b**) KAS.

The graphs of the activation energy *E_α_* based on the degree of conversion α are displayed in Figure 5. The curves produced with the KAS and OFW approaches generally have a similar form. The employment of various approximation techniques to calculate the temperature integral accounts for the slight variations.

The shape of the *E_α_* curves confirms that the biomass’s lignocellulosic content makes the mechanism of AC pyrolysis extremely complex. In the range of *α* = 0.1–0.6, the activation energy for the AC sample rose with conversion before declining as the reaction progressed. In the conversion range of α = 0.5–0.8, the activation energy peaked. A low *E_α_* was needed to break weak bonds and eliminate volatile molecules at the start of the pyrolysis process. Consequently, greater activation energy was needed to break stronger bonds to break down big, stable molecules [19,48]. With average *E_α_* values of 262.02 kJ/mol (134.71–388.44) and 254.08 kJ/mol (127.79–378.67 kJ/mol) for AC pyrolysis using the OFW and KAS techniques, respectively, the biomass was found to have high thermal stability, meaning that a substantial amount of energy was needed for the chemical reaction to take place. When amaranth inflorescences were exposed by pyrolysis, *E_α_* curves of a similar type were produced [12].

The higher initial *E_α_* (compared to that of AC) and significant peak of the blend should be explained. The higher initial *E_α_* (AC/LDPE) is likely due to the reduced reaction kinetics of AC as a result of the dilution of AC by the nonpolar polyethylene melt. The significant peak starts at the conversion of 0.2 (corresponding to 0.4 for AC). At this point, the fast decomposition of AC is approaching the end, and the decomposition of LDPE begins to dominate. However, the radical process of LDPE decomposition is inhibited by the radical scavengers in AC (lignin residues), resulting in reduced reaction kinetics for LDPE. As the scavengers were gradually consumed, the *E_α_* peak comes to an end at the conversion of 0.5. The second *E_α_* peak of AC is not observed in the *E_α_* plot of the blend. A likely explanation for the disappearance is that the radicals from polyethylene decomposition facilitate the decomposition of AC residue after the conversion of 0.5 or ~490 °C by changing the mechanism of the decomposition.

The average *E*_α_ values for LDPE pyrolysis by the OFW and KAS methods were 212.74 kJ/mol (155.23–228.89) and 200.33 kJ/mol (143.92–216.99 kJ/mol), respectively. The average activation energy values for the pyrolysis of the AC/LDPE blend by the OFW and KAS methods were 301.39 kJ/mol (207.20–481.28) and 287.69 kJ/mol (194.95–457.75 kJ/mol), respectively. The activation energies computed from the KAS and OFW methods imply the existence of complex multi-reaction mechanisms in the pyrolysis process.

#### 2.4.2. Relationship Between Kinetic Parameters

The kinetics of the pyrolysis of the mixture were studied by the CR method using theoretical forms of *g*(*α*) [26] in the range of degrees of conversion *α* = 0.2–0.8. The *E_α_* values calculated by the OFW method were used as a basis. The *E_α_* values were 332.40, 300.39, and 306.00 kJ/mol for the heating rates of 5, 10, and 20 °C/min, respectively. Table 4 presents the obtained kinetic triplet values (*g*(*α*), *E_α_*, and *A*). The obtained reaction order value in this study is comparable with those obtained for soybean straw (*n* = 8.2–17.3) [49], hazelnut husk (*n* = 12) [50], and sunflower husk (*n* = 8) [26].

**Figure 6:**
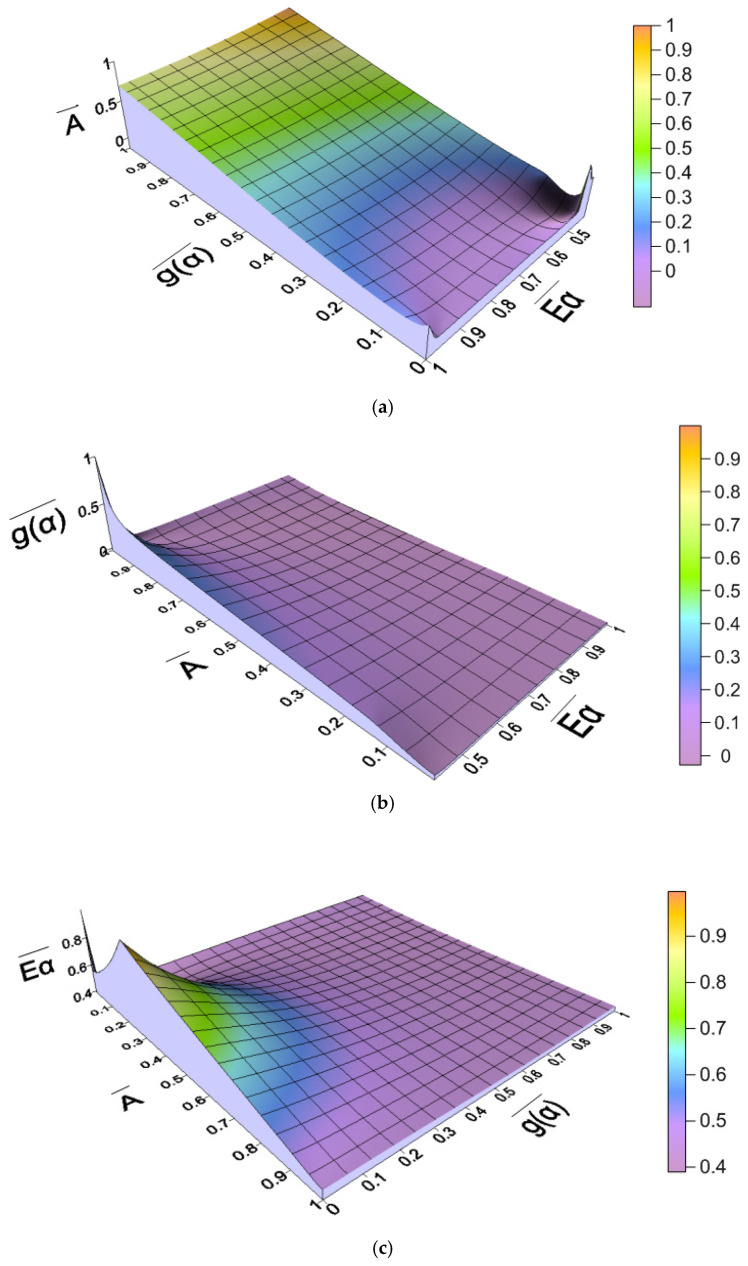
Surface plots of parameters: (**a**) pre-exponential factor; (**b**) algebraic function *g*(*α*); (**c**) activation energy.

**Figure A3:**
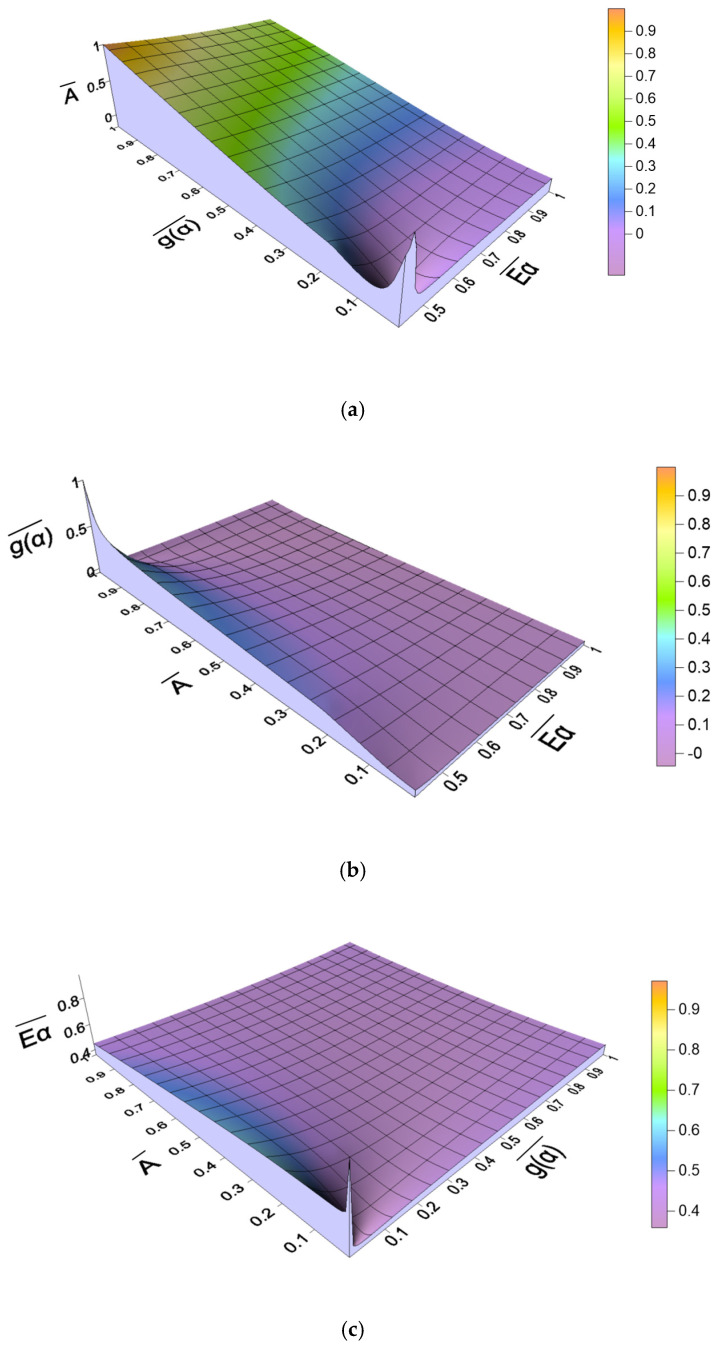
Surface plots of parameters: (**a**) pre-exponential factor; (**b**) algebraic function *g*(*α*); (**c**) activation energy under 5 °C/min.

**Figure A4:**
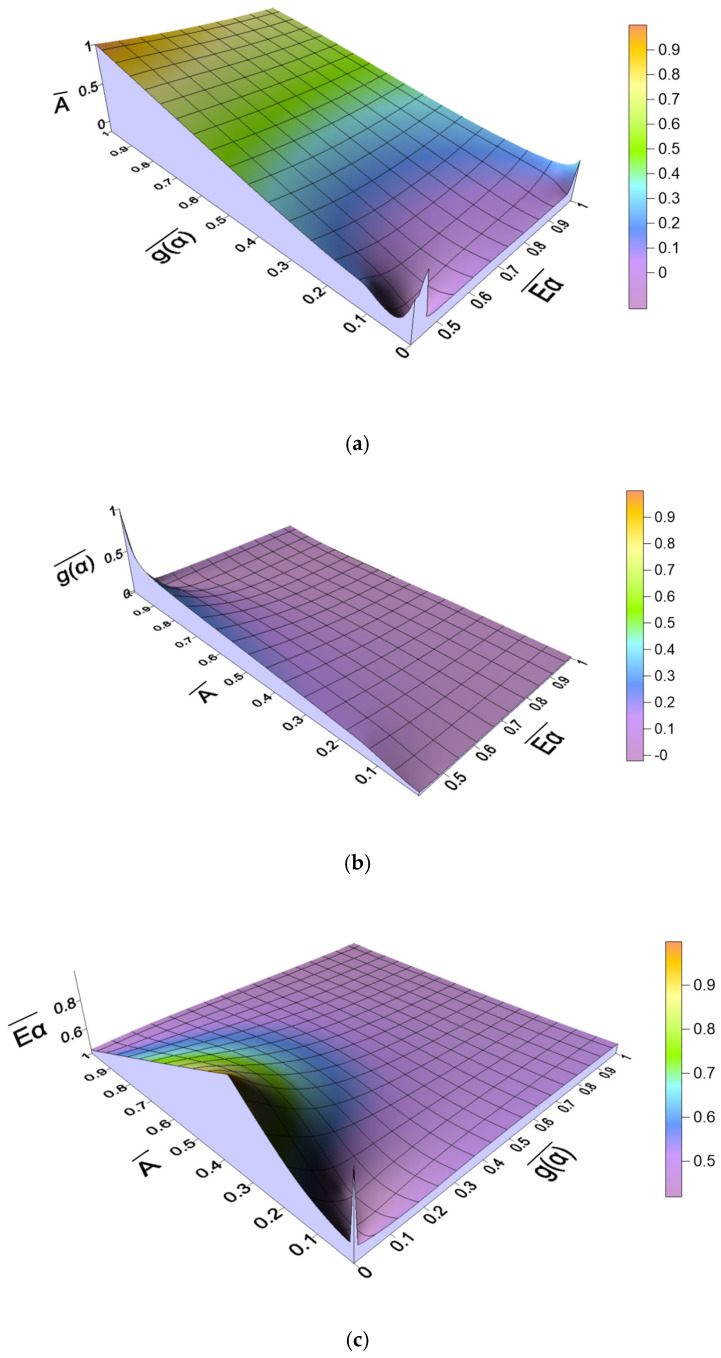
Surface plots of parameters: (**a**) pre-exponential factor; (**b**) algebraic function *g*(*α*); (**c**) activation energy under 20 °C/min.

A visual examination of the correlations of the kinetic parameters of AC/LDPE was carried out at a heating rate of 10 °C/min using the Kriging method [26]. The absolute and normalized values of the kinetic parameters are presented in Table 5. The normalized values are plotted in Figure 6, Figure A3 and Figure A4 for comparative analysis. The normalization of the parameters allows for a comparative analysis, which is usually not possible when using absolute values.

When the parameter $\bar{E_{\alpha }}$ reaches its maximum value of one, the dimensionless parameter $\bar{A}$ will also have a value of ≈0.23. The second extreme value of $\bar{E_{\alpha }}$ corresponds to the minimum value of $\bar{A}$. This is a very interesting result, as it indicates that at this point, the number of molecules with effective collisions becomes minimal, while a very large amount of energy is needed to activate the molecules.

### 2.5. Material Balance and Pyrolysis Products

#### 2.5.1. The Material Balance of the Process

**Figure 7:**
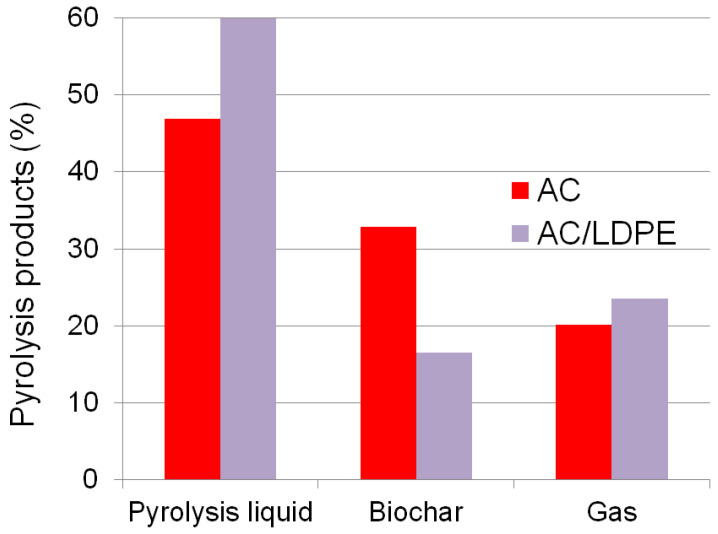
The material balance of the pyrolysis process.

To study the mass balance of the pyrolysis process, AC/LDPE blend and AC biomass samples were used. Pyrolysis liquid, solid carbonaceous residue (biochar), and pyrolysis gas were the end products. In Figure 7, the material balance is displayed.

The major portion of the material balance is made up of the pyrolysis liquid. The yields of pyrolysis liquid (up 21.72%) and pyrolysis gas (up 14.33%) both rise with the combined pyrolysis of AC and LDPE. The high concentration of volatile components in LDPE explains why the percentage of biochar drops when plastic components are added to the biomass (Table 1). The material balance of the pyrolysis of rod-milled wheat straw is similar to that of the AC sample: 46.16% pyrolysis liquid, 30.25% biochar, and 23.59% gas [51]. The biochar yield is comparable to the data obtained from the joint pyrolysis of pine bark and wheat straw with Tetra Pak waste in a mass ratio of 1:1 [52]. Tetra Pak consists of 70% paperboard, 25% LDPE, and 5% Al foil. Tetra Pak waste was cut into small square or rectangular pieces (maximum dimensions 1 mm × 2 mm). The mass of biochar with the addition of Tetra Pak waste is only 19.8% of the original mass. During the pyrolysis of pine bark, the biochar yield was about 35%, and during the pyrolysis of wheat straw, it was 26%. Therefore, the higher the content of plastic waste in the co-pyrolytic mixtures, the smaller the mass of the resulting coal.

#### 2.5.2. Composition of Pyrolysis Liquid

**Figure 8:**
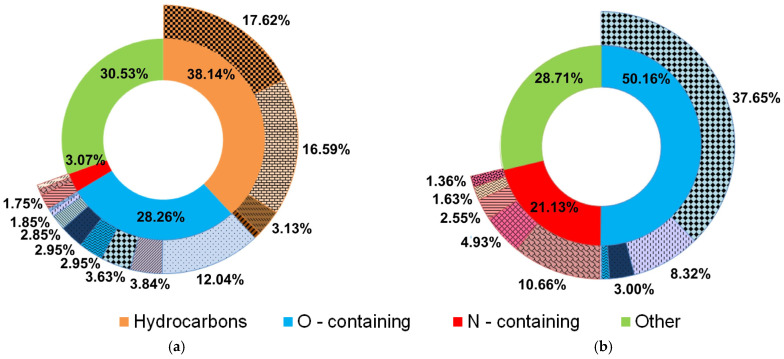
Chemical composition of pyrolysis liquid: (**a**) AC and (**b**) AC/LDPE (peak area ≥ 1%). 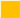 Hydrocarbons; 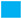 O-containing; 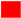 N-containing; 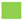 Other; 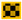 Saturated; 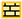 Unsaturated; 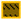 Aromatics; 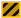 Cyclics; 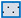 Ethers; 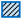 Ketones; 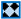 Carboxylic acids; 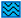 Phenols; 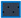 Cyclics; 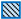 Alcohols; 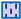 Heterocycles; 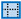 Aldehydes; 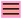 Amides; 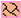 Heterocycles; 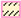
Nitriles; 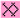 Amines; 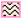 Cyclics; 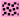 Ketones.

The pyrolysis liquid obtained from the AC sample consisted of an organic aqueous phase and a sticky phase (tars and waxes). As a result of the GC-MS analysis of the aqueous fraction of the AC sample, the mass spectra of 69 organic substances were obtained, which were combined into three groups (Figure 8a).

Hydrocarbons make up 38.14% of all detected chemicals, with aliphatic hydrocarbons accounting for the bulk (17.62% saturated and 16.59% unsaturated), with a minor proportion of aromatic (3.13%) and cyclic (0.8%) hydrocarbons. The cyclic compounds (2.85%), carboxylic acids (3.63%), alcohols (1.85%), aldehydes (0.4%), ketones (3.84%), phenols (2.95%), carboxylic acid esters (12.04%), and heterocyclic oxygen-containing compounds (0.7%) make up the majority of the group of oxygen-containing components. Nitrogen-containing chemicals, which include amides (1.75%), heterocyclic nitrogen-containing compounds (0.74%), and nitriles (0.58%), make up the smallest portion (3.07%). The presence of hydrocarbons in the pyrolysis liquid demonstrates its potential application in the biofuel industry, and an improvement in the pyrolysis process can further improve its quality and yield.

**Figure 9:**
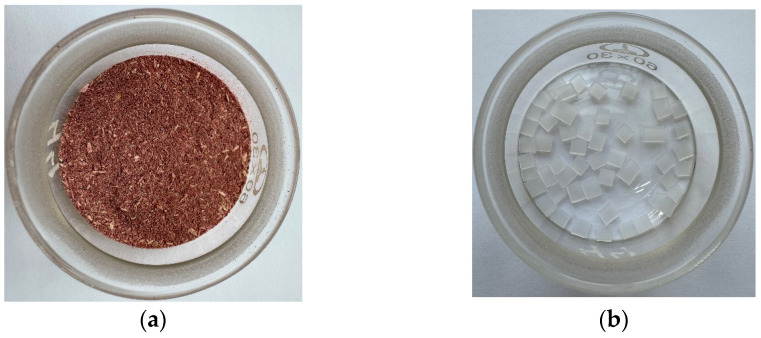
Photos of original samples: (**a**) AC; (**b**) LDPE.

The pyrolysis liquid from the AC/LDPE mixture consisted of three fractions: an organic aqueous phase, resinous phase, and a sticky phase (tars and waxes). Similar results were obtained during the combined pyrolysis of pine bark and wheat straw with Tetra Pak waste [52]. As a result of the GC-MS analysis of the organic aqueous phase, 34 organic compounds were identified (Figure 9b). It should be noted that hydrocarbons were not detected. The maximum mass fraction corresponds to oxygen-containing compounds, in particular, carboxylic acids (37.65%). Thus, hydrocarbons are found in the resinous and waxy organic fractions of the pyrolysis liquid. The light fraction, abundant in oxygenated compounds (50.16%), could certainly be further separated, and value-added chemicals could be obtained, or it could be utilized as raw material in resin making, allowing bio-based polymers to be produced. The recovery of acids such as acetic acid and n-hexadecanoic acid is possible for lighter water- and acid-rich fractions. The resinous and waxy fraction is in turn rich in hydrocarbons and low in problematic oxygenated compounds and water, thus making it suitable for fuel oil and possibly requiring less upgrading, thus lowering costs.

#### 2.5.3. Characteristics of Biochar

The primary constituents of biochar, a solid carbonaceous pyrolysis product, are carbon and minerals. The composition and chemical makeup of the organic and inorganic components that make up its matrix determine its characteristics. According to an analysis of the produced biochar, during pyrolysis, the amount of carbon and nitrogen rose, while the amount of hydrogen and oxygen fell (Table 6). The breakdown of light organic components, which release light hydrocarbons and simple-structured polymers, can account for this shift in the elemental makeup [53]. Biochar can be a helpful tool for promoting nitrogen entry into the soil–plant system, even if it has a low nitrogen content [54].

The volatile matter content of the produced biochar significantly decreased (19.3–24.6%). The thermal breakdown of flammable non-carbon components is the cause of this decline [55]. In comparison to the AC sample, the mixture’s fixed carbon content rose by 10%. In line with earlier findings, the biochar sample’s ash content increased to 27.65–31.34%, showing the concentration of inorganic components throughout the pyrolysis process [31]. K and Ca were the primary constituents of ash, accounting for 85.5% and 85.3% of the total mineral content, respectively (Table 7). Biochar’s higher potassium and calcium content, combined with magnesium, can neutralize acidic soils and boost crop development and yield by acting as a lime fertilizer [56].

The content of inorganic phosphorus in biochar is low due to the lignocellulosic composition of the biomass [57,58]. There is the suggestion that lower O:C ratios result in more stable biochar material. When the molar O:C ratio is under 0.2, the resulting biochar will possess a half-life of greater than 1000 years. Thus, after pyrolysis, the biochar has a structural arrangement of aromatic rings that create highly stable crystalline graphite-like structures. Since biochar has a low C/O ratio (0.14 and 0.1), it is expected to have a stronger graphite-like structure. Thus, biochar is proposed to be used for soil application; the physicochemical characteristics of the obtained biochar have proven its suitability for soil application.

## 3. Discussion

The search for large-tonnage renewable raw materials for obtaining high-quality energy resources is an urgent task. Biomass has enormous potential, since its resources are vast and diverse. Pyrolysis allows for the production of a full range of products, including gaseous, liquid, and solid products. Gas is most often used for in-house technological needs, so pyrolysis liquid and biochar are of particular interest. Combining the raw materials used in thermochemical conversion or co-pyrolysis allows products of the required quality to be obtained. The thermochemical conversion of two types of waste can be more effective than the use of monosubstrates [59].

The object of our study is the biomass of amaranth, a unique plant. It belongs to the C4 class, so it is able to carry out photosynthesis even at high temperatures, effectively saving water and very quickly absorbing carbon dioxide. Some species of amaranth are cosmopolitan plants, capable of growing in any climate zone, including the cryolithic zone [60]. The plant can reach 1.5–3 m in height; thus, it is possible to obtain many biomass resources with little water and fertilizer consumption [61]. Amaranth seeds can be used to produce gluten-free flour or oil with high squalene content, and the remaining waste can be sent for co-pyrolysis. Unlike many studies that analyzed biomass growing in a specific location and climate conditions, which excludes the possibility of its large-scale application worldwide, our results take this factor into account, which allows us to recommend the use of this technology everywhere.

Plastic waste is a synthetic material obtained from crude oil. It contains only carbon and hydrogen. During the pyrolysis of such raw materials, their polymeric macromolecular structures are broken down into small molecules or monomers. Accordingly, the liquid formed during thermochemical conversion is similar to conventional fuel. Thus, the addition of plastic during the pyrolysis of biomass can have a desirable effect on the composition of pyrolysis liquid [59]. Our previous studies demonstrated the possibility of obtaining a hydrocarbon-rich pyrolysis liquid from two types of amaranth and various parts of the plant (non-food), which confirms the assumption that the co-pyrolysis of amaranth biomass with plastic waste will significantly improve the composition of the pyrolysis liquid [12,31].

As a result of the pyrolysis of the AC sample, the aqueous fraction contained 38.14% hydrocarbons, and during the pyrolysis of the mixture, all hydrocarbons passed into the resinous fraction. This indicates the need to revise not the proportions of the mixed raw materials but the technology of thermochemical conversion itself. In the future, the two-stage catalytic processing of the raw materials is planned. In [62], an analysis of the pyrolysis of various biomass was carried out, as a result of which the maximum amount of certain hydrocarbons in the pyrolysis liquid was 2% [63]. Only with the hydrothermal liquefaction of *Nostoc ellipsosporum* was 25% of hydrocarbons obtained in bio-oil [64], and for the catalytic hydrothermal liquefaction of Rice straw, this value was up to 36% [65]. Thus, the obtained results demonstrate the uniqueness of the considered biomass and the mixture obtained with it. Consequently, according to the above hypothesis, the addition of plastic waste will enrich the resulting pyrolysis liquid, due to its chemical composition and high content of volatile components.

An important pyrolysis product is biochar. The addition of plastic will have a positive effect on the quantity and quality of the resulting solid product [66]. As most studies recommend the use of biochar as a soil amendment, it should be noted that biochar production has many economic and social benefits, including climate change mitigation, energy source, soil improvement, water purification, and waste management [67].

The pyrolysis of such a mixture requires a special approach to the design of the pyrolysis reactor. It is essential to better understand the kinetics of the chemical reactions occurring during the thermochemical conversion of solid waste and their mixtures in order to determine safe conditions for carrying out exothermic reactions [68]. TGA and kinetic analysis data indicate the complexity of the pyrolysis process of AC and AC/LDPE, accompanied by many sequential and parallel reactions. High variability in *E_α_* values was obtained for AC and the AC/LDPE mixture, which is explained by the multistage nature of the process [69]. This is a multicomponent biomass; therefore it is characterized by a multistage thermal decomposition process controlled by more than one energy barrier. Our results, combined with the observations of other authors, emphasize that the reaction mechanism characteristic of the thermochemical conversion process of both biomass and substrate mixtures with biomass is complex [70,71]. It can be described by an *n*-th-order reaction (*n* = 8 and *n* = 11). It should be noted that when describing the thermochemical conversion process, the pseudo-order *n* has no physical meaning, but it plays an important role in determining the reaction mechanism as a correlation parameter of the model [49,72]. This result is consistent with the results of other studies obtained in the thermochemical processing of palm oil waste (*n* = 7) [73], bean straw and corn cobs (*n* = 9–10) [74], soybean straw (*n* = 8.2–17.3) [49], and hazelnut husks (*n* = 12) [50]. A pre-exponential factor above 10^9^ 1/s indicates a complex reaction [75].

The correlation between the main parameters of the “kinetic triplet” was previously studied by us for the combustion process of sunflower pellets [26]. It should be noted that the reaction mechanism of the thermochemical conversion process is described by an 8th-order reaction, and the pre-exponential factor is also higher than 10^9^ 1/s, while the correlations between the kinetic parameters do not coincide. During combustion, the parameters ${E_{\alpha }}^{\max}$ and $A^{\max}$ correspond to *α* = 0.55, and during pyrolysis, ${E_{\alpha }}^{\max}$ is observed at *α* = 0.25 and $A^{\max}$ at *α* = 0.8. Changes in $\bar{E_{\alpha }}$ and $\bar{A}$ have different natures and are not comparable. The obtained results indicate the similarity of the change in the parameter $\bar{g\left(\alpha \right)}$. Therefore, further studies are needed to analyze the correlations between the kinetic parameters characterizing the thermochemical conversion.

## 4. Materials and Methods

In this paper, experimental studies of the pyrolysis process are carried out in a laboratory experimental setup, as well as using thermogravimetry, FTIR spectroscopy, gas chromatography, and mass spectrometry (TG-FTIR-GC-MS). To evaluate the kinetic analysis, the OFW, KAS, and Coats–Redfern methods were used to determine the parameters of the “kinetic triplet”. The results of experiments using these methods will be presented in the form of three-dimensional surfaces displaying the relationship between the normalized values of the activation energy, pre-exponential factor, and algebraic function of the reaction mechanism. The physicochemical characteristics of biochar and pyrolysis liquid will allow us to evaluate the possibility of their use as high-added-value products.

### 4.1. Sample Materials

The object of this study was the above-ground part of *A. cruentus* (AC). The samples were collected from the field, where the plant grew together with other cultivated herbs. Plants with leaves, stems, and inflorescences were cut and then dried at room temperature. The seeds were removed from the inflorescences. Dry AC biomass was crushed and sieved (Figure 9a). Food packaging waste was used to obtain an LDPE sample. It was manually cut into small pieces of 3 by 3 mm (Figure 9b, for pyrolysis) and ground to a powder state with a notched metal rod (for TGA). For the co-pyrolysis study, AC and LDPE were uniformly mixed in a mass ratio of 1:1. The sample was named AC/LDPE. All crushed samples were stored in airtight containers for use in further experiments.

### 4.2. Physicochemical Characterization

The ultimate analysis was carried out in a Euro EA3000 elemental analyzer (Eurovector, SpA, Milan, Italy). The oxygen content (*O*, wt.% on air dry basis) and the higher heating value (*HHV*) of the samples were calculated using standard formulas [31]. Proximate analysis was performed according to standards (ASTM E1755-01, ASTM E1756-08, GOST R 56881-2016, and GOST 32990-2014 ) using a drying chamber (ShSL-43/250 V, AnalytPromPribor, Moscow, Russia) and a muffle furnace (PMLS-2/1200, Milaform, Kazan, Russia). The macro- and microelement contents of the biochar samples were identified using an energy-dispersive X-ray fluorescence spectrometer (EDX-800HS2, Shimadzu, Kyoto, Japan).

The pyrolysis liquid was subjected to chromatography–mass spectrometry using an HP-5MS column (0.25 μm, 30 m, Shimadzu, Kyoto, Japan) and a spectrometer (GCMS-QP2010, Shimadzu, Kyoto, Japan). The mass spectra of the products were compared with the NIST 2020 library, and the compounds with the highest similarity were considered. This study reported the bio-oil composition as GC-MS peak area percentages, and compounds with areas higher than 1% were reported. The peak area values indicate a given compound’s quantity, and the relative peak areas show the relative content in the product’s composition.

The volatile matter (*VM*) and ash content of biochar were determined according to ASTM D3175-89 and ASTM D3174-04 using a muffle furnace (PMLS-2/1200, Milaform, Kazan, Russia). Fixed carbon (*FC*) was calculated from the difference using Equation (1):

(1) FC=100−VM−Ash

The *HHV* (MJ/kg) of the biochars was determined using Equation (2) [35]:

(2) $$HHV_{biochar}=\left(15.59\cdot VM+35.36\cdot FC-0.78\cdot Ash\right)/100$$

### 4.3. Thermogravimetric Analysis

TGA was performed using an STA 449 A1 Jupiter synchronous microthermal analyzer (Netzsch, Selb, Germany) similar to that used in [76]. All measurements were performed in a dynamic inert atmosphere (argon). A desired amount of AC (16 mg), LDPE (9 mg), and AC/LDPE (5 mg/5 mg) was taken in a crucible and placed in a TGA furnace. Three heating rates of 5, 10, and 20 °C/min were investigated during the experiments. The volatile substances released during pyrolysis were analyzed in real time by FTIR spectroscopy in a Tensor 27 FTIR spectrometer (Bruker Corp., Billerica, MA, USA). The repeatability error of the experiment corresponded to 1.5%.

### 4.4. Kinetic Analysis

The technique for performing kinetic analysis is presented in [12]. Two integral isoconversion methods, Ozawa–Flynn–Wall (OFW) and Kissinger–Akahira–Sunose (KAS), were used to evaluate the kinetic parameters of the co-pyrolysis process. According to the ICTAC kinetics committee’s recommendations [77,78], the kinetic parameters were calculated at a conversion of *α* = 0.1–0.9 with a 0.1 interval.

The Coats–Redfern (CR) model is an integral model generally used for the calculation of kinetic factors. The pre-exponential factor *A*, activation energy *E_α_*, and algebraic function *g*(*α*) are referred to as the “kinetic triplet,” which describes a specific reaction. In this study, the CR model was used to analyze the pyrolysis process of the AC/LDPE sample. To analyze the correlations of the kinetic parameters characterizing the pyrolysis process, the data is normalized in the range [0, 1]:

(3) $$\bar{E_{\alpha }}=\frac{E_{\alpha }}{{E_{\alpha }}^{\max}}$$

(4) $$\bar{A}=\frac{A}{A^{\max}}$$

(5) $$\bar{g\left(\alpha \right)}=\frac{g\left(\alpha \right)}{g{\left(\alpha \right)}^{\max}}$$

where *E_α_*, *A*, and *g*(*α*) are the absolute values of the parameters; ${E_{\alpha }}^{\max}$, $A^{\max}$, and $g{\left(\alpha \right)}^{\max}$ are the maximum value parameters.

Next, using the Kriging method, surfaces $S_{\bar{E_{\alpha }}}$, $S_{\bar{A}}$, and $S_{\bar{g\left(\alpha \right)}}$ are constructed. The details of the method used are described in [26].

### 4.5. Experimental Pyrolysis Procedure

Pyrolysis was carried out in a metal tubular reactor that was 280 mm long and had a 34 mm inner diameter (Institute of Power Engineering and Advanced Technologies, Moscow, Russia). A 20 g sample weighed to the nearest 0.0001 g was loaded into the reactor; then the reactor was placed in the furnace, and the reactor outlet pipe was connected to the condensation system. The initial temperature was an ambient temperature of 25 °C, the heating rate to reach the pyrolysis temperature was 10 °C/min, and the final heating temperature was 550 °C. Temperature variations were tracked and recorded using a thermocouple that was connected to software. The thermocouple voltage was then monitored and recorded by the data logging program, allowing for the exact tracking of temperature changes over time. The pyrolysis process’s liquid and gaseous byproducts went through a condenser. Non-condensable gas was collected in a gas bag (connected to the outflow of the liquid collecting flask), and the pyrolysis liquid was collected in a flask. After cooling, the solid residue that was still in the reactor was weighed. Samples of AC and AC/LDPE were examined. Each experiment was carried out for 120 min. All the experiments were repeated thrice, and average data is reported.

The material balance of the pyrolysis process was calculated using the following formulas:

(6) $$W_{biochar}=\frac{W_{2}-W_{1}}{W_{feed}}\times 100$$

(7) $$W_{liquid}=\frac{W_{4}-W_{3}}{W_{feed}}\times 100$$

(8) $$W_{gas}=100-\left(W_{liquid}+W_{carb.r}\right)$$

where *W_feed_* is the weight of the sample (g); *W*_1_ is the mass of the empty reactor before pyrolysis (g); *W*_2_ is the mass of the reactor after pyrolysis (g); *W*_3_ is the mass of the empty flask (g); *W*_4_ is the mass of the flask with the liquid product (g); *W_biochar_* is the yield of biochar (wt.%); *W_liquid_* is the yield of pyrolysis liquid (wt.%); *W_gas_* is gas yield (wt.%).

## 5. Conclusions

This study examined the thermal degradation of amaranth residue and waste LDPE utilizing a slow pyrolysis reactor in the lab and the TG-FTIR-GC MS method. Three steps were evident in the AC/LDPE sample, which is typical of the lignocellulosic feedstock’s thermal breakdown. According to the OFW and KAS techniques, the average activation energies for the pyrolysis of AC/LDPE were 287.69 kJ/mol and 301.39 kJ/mol, respectively. The normalization of kinetic parameters allows us to represent the “kinetic triplet” as a plane, which significantly simplifies the procedure for studying chemical reactions occurring during thermochemical conversion. Using this analysis method, it is possible to compare various thermal conversion processes not only of biomass but also of other materials. During the pyrolysis of the AC/LDPE mixture, hydrocarbons were found in the resinous and waxy organic fractions of the pyrolysis liquid. Thus, it is necessary to study not only different proportions of AC and LDPE but also another technology of the pyrolysis process itself. Biochar can be used as a useful agricultural product.

## Figures and Tables

**Table 1:** Proximate and ultimate analyses of samples.

Samples	Ultimate Analysis (wt.%, on Air Dry Basis)				Proximate Analysis (wt.%, on Air Dry Basis)				HHV (MJ/kg)
	C	H	N	O	Moisture	Ash	Volatile Matter	Fixed Carbon	
AC	38.49 ± 0.07	6.09 ± 0.3	1.44 ± 0.5	40.98 ± 1.02	7.4 ± 0.03	13.0 ± 0.7	75.8 ± 0.9	11.2 ± 0.2	20.3 ± 0.47
LDPE	85.43 ± 0.5	13.52 ± 0.6	-	0.95 ± 0.9	0.0	0.1 ± 0.02	99.9 ± 0.4	0.0	45.7 ± 0.7
AC/LDPE	61.96 ± 0.9	9.81 ± 0.09	0.72 ± 0.02	21.01 ± 0.1	3.7 ± 0.07	6.5 ± 0.4	87.9 ± 1.02	5.6 ± 0.3	31.0 ± 0.42

**Table 2:** Main stages of AC/LDPE thermal decomposition.

Pyrolysis Stage	Starting Temperature (°C)	Ending Temperature (°C)	Temperature Peak on DTG Curve (°C)
Drying	25	190	95
Fast decomposition	190	550	467
Slow decomposition	550	1000	-

**Table 3:** Results of TG analysis of samples.

Samples	Mass Loss (wt.%, on Air Dry Basis)			
	Drying	Fast Decomposition	Slow Decomposition	Residual Mass at 1000 °C
AC	8.3	64.9	7.8	27.3
LDPE	0.0	92.7	0.01	7.12
AC/LDPE	2.3	76.8	5.1	18.1

**Table 4:** Average values of AC/LDPE triplet parameters for *α* = 0.2–0.8 and linear regression equations.

*β* (°C/min)	Reaction Mechanism	Linear Regression Equation	*g*(*α*)	*E_α_* (kJ/mol)	*A* (1/s)
5	F_9_	y = −33.54x + 39.98	36,803.89	332.40	5.31 × 10^27^
10	F_11_	y = −25.34x + 30.08	848,768.02	300.39	2.23 × 10^26^
20	F_11_	y = −25.63x + 30.06	848,768.02	306.00	3.28 × 10^26^

**Table 5:** Parameters of AC/LDPE kinetic triplet (CR method).

α	$E_{\alpha }\left(\mathrm{kJ}/\mathrm{mol}\right)$	$\bar{E_{\alpha }}$	*A* (1/s)	$\bar{A}$	$g\left(\alpha \right)$	$\bar{g\left(\alpha \right)}$
0.20	254.39	0.45	1.19 × 10^27^	0.99	8.31	0.00
0.25	**564.39**	**1.00**	2.82 × 10^26^	0.23	16.8	0.00
0.30	481.28	0.85	1.07 × 10^24^	0.00	34.4	0.00
0.35	551.35	0.98	1.85 × 10^23^	0.00	73.3	0.00
0.40	436.12	0.77	2.02 × 10^23^	0.00	164	0.00
0.45	322.35	0.57	2.97 × 10^23^	0.00	394	0.00
0.50	294.43	0.52	5.46 × 10^23^	0.00	1020	0.00
0.55	270.83	0.48	1.28 × 10^24^	0.00	2940	0.00
0.60	261.61	0.46	3.17 × 10^24^	0.00	9540	0.00
0.65	253.06	0.49	9.21 × 10^24^	0.01	36,200	0.00
0.70	245.40	0.44	3.30 × 10^25^	0.03	169,000	0.02
0.75	247.26	0.44	1.68 × 10^26^	0.14	1,050,000	0.11
0.80	240.13	0.43	**1.21** × 10^27^	**1.00**	**9,770,000**	**1.00**

**Table 6:** The results of the analyses for biochars.

Samples	Ultimate Analysis (wt.%, on Air Dry Basis)				Proximate Analysis (wt.%, on Air Dry Basis)			HHV, MJ/kg
	C	H	N	O	Ash	Volatile Matter	Fixed Carbon	
AC_biochar	57.67 ± 1.07	2.30 ± 0.25	1.92 ± 0.6	10.46 ± 0.7	27.65 ± 0.1	24.6 ± 0.3	47.75 ± 0.8	18.9 ± 0.15
AC/LDPE_biochar	57.16 ± 0.9	1.97 ± 0.2	1.87 ± 0.02	7.66 ± 0.3	31.34 ± 0.06	19.3 ± 0.8	49.36 ± 0.5	19.7 ± 0.06

**Table 7:** Chemical composition of mineral part of biochar.

Samples	Content of Macro- and Microelements (wt.%, on Air Dry Basis)												
	K	Ca	Mg	P	Cl	S	Si	Fe	Ti	Zn	Mn	Br	Sr
AC_biochar	49.8	35.7	4.1	3.8	2.4	1.6	1.3	0.7	0.2	0.1	0.1	0.04	0.03
AC/LDPE_biochar	52.6	32.7	3.9	3.6	2.9	1.5	1.7	0.8	-	0.1	0.1	0.04	0.04

## Data Availability

The original contributions presented in this study are included in the article. Further inquiries can be directed to the corresponding author.

## Appendix A

Figure A1 displays the samples’ and their mixture’s TG and DTG curves at a heating rate of 5 °C/min.

Figure A2 displays the samples’ and their mixture’s TG and DTG curves at a heating rate of 20 °C/min.
